# (3-Oxo-3*H*-benzo[*f*]chromen-1-yl)methyl *N*,*N*-dimethyl­carbamodithio­ate

**DOI:** 10.1107/S160053681203975X

**Published:** 2012-09-26

**Authors:** N. M. Mahabaleshwaraiah, H. R. Ravi, M. Vinduvahini, H. R. Sreepad, O. Kotresh

**Affiliations:** a Department of Chemistry, Karnatak University’s Karnatak Science College, Dharwad, Karnataka 580 001, India; b Research Centre, Postgraduate Department of Physics, Government First Grade College (Autonomous), Mandya 571 401, Karnataka, India; c Department of Physics, Sri D Devaraja Urs Government First Grade College, Hunsur 571 105, Mysore District, Karnataka, India

## Abstract

In the title compound, C_17_H_15_NO_2_S_2_, the 3*H*-benzo[*f*]chromene ring system is distinctly twisted; the dihedral angle between the pyran ring and its opposite benzene ring is 9.11 (8)°. The *N*,*N*-dimethyl­carbamodithio­ate residue lies almost perpendicular to the pyran ring [dihedral angle = 85.15 (7)°]. In the crystal, weak C—H⋯O hydrogen bonds link the mol­ecules into *C*(10) chains propagating in [001].

## Related literature

For a related structure and background to coumarins, see: Kant *et al.* (2012 ▶); For the synthesis of the title compound, see: Kumar *et al.* (2012 ▶).
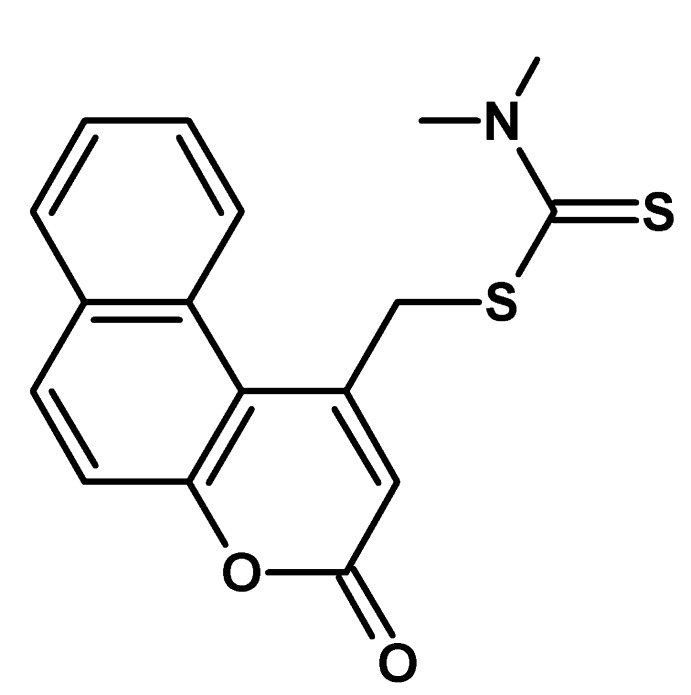


## Experimental

### 

#### Crystal data

C_17_H_15_NO_2_S_2_
*M* *_r_* = 329.42
Monoclinic, 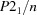

*a* = 14.1575 (2) Å
*b* = 6.9399 (1) Å
*c* = 15.9750 (2) Å
β = 101.591 (1)°
*V* = 1537.56 (4) Å^3^
*Z* = 4
Mo *K*α radiation
μ = 0.35 mm^−1^
*T* = 296 K
0.24 × 0.20 × 0.12 mm

#### Data collection

Bruker SMART CCD diffractometer
Absorption correction: multi-scan (*SADABS*; Bruker, 2001 ▶) *T*_min_ = 0.770, *T*_max_ = 1.000
14561 measured reflections
2708 independent reflections
2387 reflections with *I* > 2σ(*I*)
*R*_int_ = 0.023

#### Refinement

*R*[*F* ^2^ > 2σ(*F* ^2^)] = 0.034
*wR*(*F* ^2^) = 0.104
*S* = 1.06
2708 reflections
199 parameters
H-atom parameters constrained
Δρ_max_ = 0.28 e Å^−3^
Δρ_min_ = −0.24 e Å^−3^

### 

Data collection: *SMART* (Bruker, 2001 ▶); cell refinement: *SAINT* (Bruker, 2001 ▶); data reduction: *SAINT*; program(s) used to solve structure: *SHELXS97* (Sheldrick, 2008 ▶); program(s) used to refine structure: *SHELXL97* (Sheldrick, 2008 ▶); molecular graphics: *ORTEP-3* (Farrugia, 1997 ▶); software used to prepare material for publication: *SHELXL97*.

## Supplementary Material

Crystal structure: contains datablock(s) I, global. DOI: 10.1107/S160053681203975X/hb6942sup1.cif

Structure factors: contains datablock(s) I. DOI: 10.1107/S160053681203975X/hb6942Isup2.hkl

Supplementary material file. DOI: 10.1107/S160053681203975X/hb6942Isup3.cml

Additional supplementary materials:  crystallographic information; 3D view; checkCIF report

## Figures and Tables

**Table 1:** Hydrogen-bond geometry (Å, °)

*D*—H⋯*A*	*D*—H	H⋯*A*	*D*⋯*A*	*D*—H⋯*A*
C2—H2⋯O2^i^	0.93	2.51	3.405 (3)	162

Symmetry code: (i) 
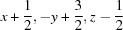
.

**Fig. 1.**
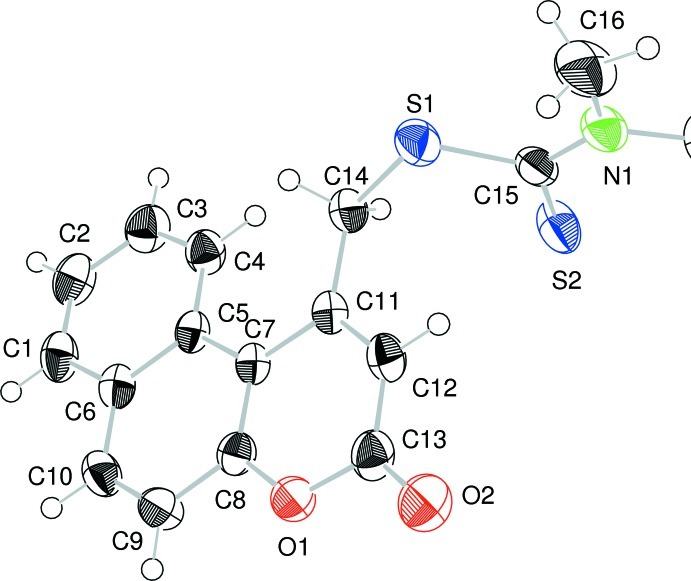
The molecular structure of the title compound. Displacement ellipsoids are drawn at the 50% probability level. The H atoms are shown as spheres of arbitrary radii.

**Fig. 2.**
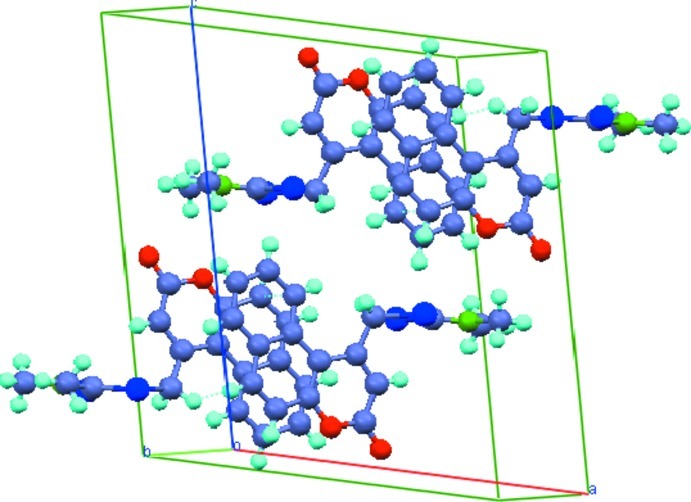
Packing of the molecules.

## supplementary crystallographic information

### Experimental

The title compound was synthesized according to the reported method (Kumar *et al.*, 2012). It was recrystallized from an ethanol–chloroform solvent mixture as colourless plates. Yield = 81%, m.p. 435 K.

### Refinement

All H atoms were positioned geometrically, with C—H = 0.93 Å for aromatic H, C—H = 0.97 Å for methylene H and C—H = 0.96 Å for methyl H, and refined using a riding model with *U*_iso_(H) = 1.5*U*_eq_(C) for methyl H and *U*_iso_(H) = 1.2*U*_eq_(C) for all other H.

### Crystal data

**Table:**

C_17_H_15_NO_2_S_2_	*F*(000) = 688
*M**_r_* = 329.42	*D*_x_ = 1.423 Mg m^−^^3^
Monoclinic, *P*2_1_/*n*	Melting point: 435 K
Hall symbol: -P 2yn	Mo *K*α radiation, λ = 0.71073 Å
*a* = 14.1575 (2) Å	Cell parameters from 2708 reflections
*b* = 6.9399 (1) Å	θ = 1.8–25.0°
*c* = 15.9750 (2) Å	µ = 0.35 mm^−^^1^
β = 101.591 (1)°	*T* = 296 K
*V* = 1537.56 (4) Å^3^	Plate, colourless
*Z* = 4	0.24 × 0.20 × 0.12 mm

### Data collection

**Table:**

Bruker SMART CCD diffractometer	2708 independent reflections
Radiation source: fine-focus sealed tube	2387 reflections with *I* > 2σ(*I*)
Graphite monochromator	*R*_int_ = 0.023
ω and φ scans	θ_max_ = 25.0°, θ_min_ = 1.8°
Absorption correction: multi-scan (*SADABS*; Bruker, 2001)	*h* = −16→16
*T*_min_ = 0.770, *T*_max_ = 1.000	*k* = −8→8
14561 measured reflections	*l* = −18→18

### Refinement

**Table:**

Refinement on *F*^2^	Primary atom site location: structure-invariant direct methods
Least-squares matrix: full	Secondary atom site location: difference Fourier map
*R*[*F*^2^ > 2σ(*F*^2^)] = 0.034	Hydrogen site location: inferred from neighbouring sites
*wR*(*F*^2^) = 0.104	H-atom parameters constrained
*S* = 1.06	*w* = 1/[σ^2^(*F*_o_^2^) + (0.0638*P*)^2^ + 0.371*P*] where *P* = (*F*_o_^2^ + 2*F*_c_^2^)/3
2708 reflections	(Δ/σ)_max_ < 0.001
199 parameters	Δρ_max_ = 0.28 e Å^−^^3^
0 restraints	Δρ_min_ = −0.24 e Å^−^^3^

### Special details

**Table:**

Experimental. IR (KBr): 660 cm^-1^ (C—S), 1251 cm^-1^ (C═S), 1036 cm^-1^ (C—O), 842 cm^-1^ (C—N), 1279 cm^-1^ (C—O—C), 1708.6 cm^-1^ (C═O). GCMS: m/e: 335. 1H NMR (400 MHz, DMSO.D_6_, δ, p.p.m.): 1.92 (m, 2H, C_10_), 2.01 (m, 2H, C_1_), 2.49 (m, 4H, C_2_, *C*_11_), 3.80 (s, 3H, C_9_), 4.86 (s, 2H, C_4_), 6.57 (s, 1*H*, C_12_), 7.24 (m, 1*H*, C_15_), 7.36 (t, 1*H*, C_7_), 7.38 (s, 1H, C_16_). Elemental analysis: C, 57.26; H, 5.07; N, 4.15.
Geometry. All s.u.'s (except the s.u. in the dihedral angle between two l.s. planes) are estimated using the full covariance matrix. The cell s.u.'s are taken into account individually in the estimation of s.u.'s in distances, angles and torsion angles; correlations between s.u.'s in cell parameters are only used when they are defined by crystal symmetry. An approximate (isotropic) treatment of cell s.u.'s is used for estimating s.u.'s involving l.s. planes.
Refinement. Refinement of *F*^2^ against ALL reflections. The weighted *R*-factor *wR* and goodness of fit *S* are based on *F*^2^, conventional *R*-factors *R* are based on *F*, with *F* set to zero for negative *F*^2^. The threshold expression of *F*^2^ > 2σ(*F*^2^) is used only for calculating *R*-factors(gt) *etc*. and is not relevant to the choice of reflections for refinement. *R*-factors based on *F*^2^ are statistically about twice as large as those based on *F*, and *R*-factors based on ALL data will be even larger.

### Fractional atomic coordinates and isotropic or equivalent isotropic displacement parameters (Å^2^)

**Table:**

	*x*	*y*	*z*	*U*_iso_*/*U*_eq_	
S1	−0.10475 (3)	0.58858 (7)	0.13224 (3)	0.04867 (17)	
S2	−0.13511 (3)	1.01975 (7)	0.13357 (4)	0.05409 (18)	
O1	0.15254 (9)	0.74478 (19)	0.41212 (8)	0.0436 (3)	
O2	0.01004 (11)	0.7691 (3)	0.44665 (9)	0.0652 (4)	
N1	−0.27082 (10)	0.7553 (3)	0.12911 (10)	0.0488 (4)	
C1	0.41046 (13)	0.7183 (3)	0.19340 (13)	0.0456 (4)	
H1	0.4754	0.7065	0.2185	0.055*	
C2	0.38414 (14)	0.7329 (3)	0.10721 (13)	0.0508 (5)	
H2	0.4303	0.7288	0.0733	0.061*	
C3	0.28694 (14)	0.7539 (3)	0.07027 (13)	0.0499 (5)	
H3	0.2688	0.7681	0.0113	0.060*	
C4	0.21747 (13)	0.7542 (3)	0.11888 (11)	0.0416 (4)	
H4	0.1532	0.7687	0.0921	0.050*	
C5	0.24097 (12)	0.7331 (2)	0.20858 (11)	0.0328 (4)	
C6	0.34099 (12)	0.7208 (2)	0.24588 (12)	0.0366 (4)	
C7	0.17168 (11)	0.7307 (2)	0.26443 (10)	0.0314 (3)	
C8	0.20897 (12)	0.7360 (2)	0.35158 (11)	0.0354 (4)	
C9	0.30765 (13)	0.7276 (3)	0.38766 (12)	0.0435 (4)	
H9	0.3286	0.7302	0.4467	0.052*	
C10	0.37191 (12)	0.7158 (3)	0.33586 (12)	0.0426 (4)	
H10	0.4373	0.7042	0.3595	0.051*	
C11	0.06649 (11)	0.7181 (2)	0.23967 (11)	0.0339 (4)	
C12	0.01393 (12)	0.7310 (3)	0.30092 (12)	0.0409 (4)	
H12	−0.0529	0.7268	0.2842	0.049*	
C13	0.05420 (13)	0.7508 (3)	0.38989 (12)	0.0446 (4)	
C14	0.01533 (12)	0.6860 (3)	0.14798 (11)	0.0404 (4)	
H14A	0.0544	0.5996	0.1213	0.048*	
H14B	0.0123	0.8083	0.1181	0.048*	
C15	−0.17873 (12)	0.7969 (3)	0.13185 (11)	0.0397 (4)	
C16	−0.31006 (16)	0.5603 (4)	0.12493 (16)	0.0676 (6)	
H16A	−0.3781	0.5660	0.1236	0.101*	
H16B	−0.2789	0.4888	0.1742	0.101*	
H16C	−0.2990	0.4977	0.0742	0.101*	
C17	−0.34233 (14)	0.9085 (4)	0.12752 (16)	0.0668 (6)	
H17A	−0.4046	0.8523	0.1258	0.100*	
H17B	−0.3443	0.9873	0.0778	0.100*	
H17C	−0.3249	0.9863	0.1779	0.100*	

### Atomic displacement parameters (Å^2^)

**Table:**

	*U*^11^	*U*^22^	*U*^33^	*U*^12^	*U*^13^	*U*^23^
S1	0.0357 (3)	0.0472 (3)	0.0603 (3)	−0.00403 (19)	0.0030 (2)	−0.0062 (2)
S2	0.0350 (3)	0.0487 (3)	0.0741 (4)	−0.0002 (2)	0.0003 (2)	0.0049 (2)
O1	0.0400 (7)	0.0563 (7)	0.0364 (6)	0.0031 (5)	0.0123 (5)	0.0014 (5)
O2	0.0541 (9)	0.0994 (12)	0.0492 (8)	0.0075 (8)	0.0273 (7)	0.0011 (8)
N1	0.0305 (8)	0.0658 (10)	0.0500 (9)	−0.0064 (7)	0.0073 (7)	−0.0073 (8)
C1	0.0313 (9)	0.0448 (10)	0.0642 (12)	0.0016 (7)	0.0176 (8)	0.0010 (9)
C2	0.0462 (11)	0.0542 (11)	0.0603 (12)	0.0013 (8)	0.0305 (10)	0.0005 (9)
C3	0.0536 (12)	0.0564 (11)	0.0447 (10)	0.0038 (9)	0.0214 (9)	0.0036 (9)
C4	0.0372 (9)	0.0475 (10)	0.0422 (9)	0.0035 (7)	0.0125 (8)	0.0037 (8)
C5	0.0327 (8)	0.0267 (7)	0.0406 (9)	0.0024 (6)	0.0107 (7)	0.0020 (6)
C6	0.0330 (9)	0.0291 (8)	0.0492 (10)	0.0002 (6)	0.0123 (7)	0.0009 (7)
C7	0.0294 (8)	0.0272 (7)	0.0385 (9)	0.0031 (6)	0.0090 (6)	0.0025 (6)
C8	0.0365 (9)	0.0336 (8)	0.0381 (9)	0.0015 (6)	0.0116 (7)	0.0017 (7)
C9	0.0397 (10)	0.0493 (10)	0.0393 (9)	−0.0007 (8)	0.0026 (7)	0.0006 (8)
C10	0.0291 (8)	0.0437 (10)	0.0525 (10)	0.0000 (7)	0.0025 (7)	0.0017 (8)
C11	0.0311 (8)	0.0318 (8)	0.0393 (9)	0.0039 (6)	0.0080 (7)	0.0027 (6)
C12	0.0295 (8)	0.0464 (10)	0.0476 (10)	0.0032 (7)	0.0097 (7)	0.0041 (8)
C13	0.0395 (10)	0.0511 (10)	0.0463 (10)	0.0044 (8)	0.0163 (8)	0.0042 (8)
C14	0.0305 (8)	0.0471 (10)	0.0439 (9)	0.0031 (7)	0.0082 (7)	−0.0025 (7)
C15	0.0298 (8)	0.0539 (11)	0.0334 (8)	−0.0012 (7)	0.0019 (6)	−0.0023 (7)
C16	0.0446 (12)	0.0797 (16)	0.0781 (15)	−0.0233 (11)	0.0115 (11)	−0.0075 (13)
C17	0.0313 (10)	0.0920 (18)	0.0773 (15)	0.0048 (10)	0.0113 (10)	−0.0135 (13)

### Geometric parameters (Å, º)

**Table:**

S1—C15	1.7844 (18)	C6—C10	1.416 (3)
S1—C14	1.7997 (17)	C7—C8	1.387 (2)
S2—C15	1.6637 (18)	C7—C11	1.465 (2)
O1—C13	1.367 (2)	C8—C9	1.401 (2)
O1—C8	1.374 (2)	C9—C10	1.350 (3)
O2—C13	1.207 (2)	C9—H9	0.9300
N1—C15	1.327 (2)	C10—H10	0.9300
N1—C16	1.460 (3)	C11—C12	1.346 (2)
N1—C17	1.465 (3)	C11—C14	1.514 (2)
C1—C2	1.356 (3)	C12—C13	1.428 (3)
C1—C6	1.415 (2)	C12—H12	0.9300
C1—H1	0.9300	C14—H14A	0.9700
C2—C3	1.392 (3)	C14—H14B	0.9700
C2—H2	0.9300	C16—H16A	0.9600
C3—C4	1.370 (2)	C16—H16B	0.9600
C3—H3	0.9300	C16—H16C	0.9600
C4—C5	1.412 (2)	C17—H17A	0.9600
C4—H4	0.9300	C17—H17B	0.9600
C5—C6	1.424 (2)	C17—H17C	0.9600
C5—C7	1.453 (2)		
			
C15—S1—C14	103.50 (8)	C9—C10—H10	119.6
C13—O1—C8	121.63 (14)	C6—C10—H10	119.6
C15—N1—C16	124.41 (17)	C12—C11—C7	118.71 (15)
C15—N1—C17	120.92 (18)	C12—C11—C14	119.09 (15)
C16—N1—C17	114.64 (17)	C7—C11—C14	122.18 (14)
C2—C1—C6	121.25 (17)	C11—C12—C13	124.16 (16)
C2—C1—H1	119.4	C11—C12—H12	117.9
C6—C1—H1	119.4	C13—C12—H12	117.9
C1—C2—C3	119.01 (17)	O2—C13—O1	117.55 (17)
C1—C2—H2	120.5	O2—C13—C12	126.46 (18)
C3—C2—H2	120.5	O1—C13—C12	115.97 (15)
C4—C3—C2	121.41 (19)	C11—C14—S1	116.43 (12)
C4—C3—H3	119.3	C11—C14—H14A	108.2
C2—C3—H3	119.3	S1—C14—H14A	108.2
C3—C4—C5	121.69 (17)	C11—C14—H14B	108.2
C3—C4—H4	119.2	S1—C14—H14B	108.2
C5—C4—H4	119.2	H14A—C14—H14B	107.3
C4—C5—C6	116.26 (15)	N1—C15—S2	124.15 (14)
C4—C5—C7	125.03 (15)	N1—C15—S1	113.33 (14)
C6—C5—C7	118.66 (15)	S2—C15—S1	122.51 (10)
C1—C6—C10	119.42 (16)	N1—C16—H16A	109.5
C1—C6—C5	120.26 (17)	N1—C16—H16B	109.5
C10—C6—C5	120.31 (15)	H16A—C16—H16B	109.5
C8—C7—C5	116.65 (15)	N1—C16—H16C	109.5
C8—C7—C11	115.72 (14)	H16A—C16—H16C	109.5
C5—C7—C11	127.61 (15)	H16B—C16—H16C	109.5
O1—C8—C7	123.37 (15)	N1—C17—H17A	109.5
O1—C8—C9	112.63 (15)	N1—C17—H17B	109.5
C7—C8—C9	123.98 (15)	H17A—C17—H17B	109.5
C10—C9—C8	119.30 (17)	N1—C17—H17C	109.5
C10—C9—H9	120.4	H17A—C17—H17C	109.5
C8—C9—H9	120.4	H17B—C17—H17C	109.5
C9—C10—C6	120.78 (16)		
			
C6—C1—C2—C3	1.1 (3)	C8—C9—C10—C6	3.0 (3)
C1—C2—C3—C4	−2.0 (3)	C1—C6—C10—C9	176.69 (17)
C2—C3—C4—C5	−0.1 (3)	C5—C6—C10—C9	−1.8 (3)
C3—C4—C5—C6	2.9 (3)	C8—C7—C11—C12	6.2 (2)
C3—C4—C5—C7	−179.57 (17)	C5—C7—C11—C12	−175.55 (15)
C2—C1—C6—C10	−176.61 (17)	C8—C7—C11—C14	−172.20 (15)
C2—C1—C6—C5	1.9 (3)	C5—C7—C11—C14	6.1 (2)
C4—C5—C6—C1	−3.8 (2)	C7—C11—C12—C13	−1.9 (3)
C7—C5—C6—C1	178.55 (15)	C14—C11—C12—C13	176.53 (16)
C4—C5—C6—C10	174.68 (15)	C8—O1—C13—O2	−176.03 (17)
C7—C5—C6—C10	−3.0 (2)	C8—O1—C13—C12	5.1 (2)
C4—C5—C7—C8	−171.14 (16)	C11—C12—C13—O2	177.5 (2)
C6—C5—C7—C8	6.3 (2)	C11—C12—C13—O1	−3.8 (3)
C4—C5—C7—C11	10.6 (3)	C12—C11—C14—S1	−20.2 (2)
C6—C5—C7—C11	−171.94 (15)	C7—C11—C14—S1	158.21 (12)
C13—O1—C8—C7	−0.7 (2)	C15—S1—C14—C11	86.48 (14)
C13—O1—C8—C9	−178.99 (16)	C16—N1—C15—S2	178.01 (16)
C5—C7—C8—O1	176.45 (14)	C17—N1—C15—S2	−0.1 (3)
C11—C7—C8—O1	−5.1 (2)	C16—N1—C15—S1	−1.3 (2)
C5—C7—C8—C9	−5.4 (2)	C17—N1—C15—S1	−179.39 (14)
C11—C7—C8—C9	173.06 (15)	C14—S1—C15—N1	−173.89 (13)
O1—C8—C9—C10	179.10 (16)	C14—S1—C15—S2	6.82 (14)
C7—C8—C9—C10	0.8 (3)		

### Hydrogen-bond geometry (Å, º)

**Table:**

*D*—H···*A*	*D*—H	H···*A*	*D*···*A*	*D*—H···*A*
C2—H2···O2^i^	0.93	2.51	3.405 (3)	162

Symmetry code: (i) *x*+1/2, −*y*+3/2, *z*−1/2.
